# Long‐term results of a randomized controlled trial comparing neoadjuvant Adriamycin, cisplatin, and 5‐fluorouracil vs docetaxel, cisplatin, and 5‐fluorouracil followed by surgery for esophageal cancer (OGSG1003)

**DOI:** 10.1002/ags3.12388

**Published:** 2020-11-28

**Authors:** Keijiro Sugimura, Makoto Yamasaki, Takushi Yasuda, Masahiko Yano, Motohiro Hirao, Kazumasa Fujitani, Yutaka Kimura, Hiroshi Miyata, Masaaki Motoori, Atsushi Takeno, Osamu Shiraishi, Tomoki Makino, Takayuki Kii, Koji Tanaka, Taro Satoh, Masaki Mori, Yuichiro Doki

**Affiliations:** ^1^ Department of Surgery Osaka International Cancer Institute Osaka Japan; ^2^ Departments of Gastroenterological Surgery Graduate School of Medicine Osaka University Suita Japan; ^3^ Department of Surgery Kinki University Faculty of Medicine Osaka Sayama Osaka Japan; ^4^ Department of Surgery National Hospital Organization Osaka National Hospital Osaka Japan; ^5^ Department of Surgery Osaka General Medical Center Osaka Japan; ^6^ Department of Surgery Kansai Rosai Hospital Hyogo Japan; ^7^ Cancer Chemotherapy Center Osaka Medical College Hospital Osaka Japan; ^8^ Department of Frontier Science for Cancer and Chemotherapy Osaka University Graduate School of Medicine Suita Japan; ^9^ Department of Surgery and Science Graduate School of Medical Sciences Kyusyu University Fukuoka Japan

**Keywords:** esophageal cancer, neoadjuvant chemotherapy, squamous cell carcinoma

## Abstract

**Aim:**

The aim is to report the long‐term outcomes of preoperative cisplatin and fluorouracil plus docetaxel (DCF) vs Adriamycin (ACF) for resectable esophageal squamous cell carcinoma (ESCC). Previously, this trial showed that DCF is associated with prolonged recurrence‐free survival (RFS).

**Methods:**

Patients were randomly assigned to two cycles of ACF (35 mg/m^2^ of Adriamycin, 70 mg/m^2^ of cisplatin intravenously on day 1, and 700 mg/m^2^ of fluorouracil infusion for 7 days) every 4 weeks or DCF (70 mg/m^2^ of docetaxel, 70 mg/m^2^ of cisplatin intravenously on day 1, and 700 mg/m^2^ of fluorouracil infusion for 5 days) every 3 weeks, followed by surgery. The primary endpoint was RFS. The secondary endpoint was overall survival (OS).

**Results:**

Between October 2011 and October 2013, 162 patients at 10 institutions were enrolled in the study, 162 of whom were eligible and randomly assigned to the two groups. The median follow‐up for surviving patients was 69.8 months. The 5‐year RFS was significantly better in the DCF group than in the ACF group (59.9% vs 40.7%, hazard ratio [HR] 0.55; 95% confidence interval [CI], 0.35‐0.86; *P* = .009) and the 5‐year OS was significantly better in the DCF group than in the ACF group (63.5% vs 49.4%, HR, 0.61; 95% CI, 0.38‐0.96; *P* = .03). The benefit of DCF chemotherapy on survival was significantly greater in the subgroups with more advanced clinical T and N stage.

**Conclusions:**

Cisplatin and fluorouracil plus docetaxel are associated with better RFS and OS than ACF in resectable ESCC patients.

## Graphical abstract

We updated the long‐term outcomes of the trial comparing cisplatin and fluorouracil (CF) plus docetaxel (DCF) or Adriamycin (ACF) as preoperative chemotherapy for esophageal squamous cell carcinoma (ESCC). The results showed that DCF regimen improves not only recurrence‐free survival, but also overall survival, compared to ACF.

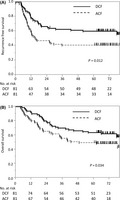

## 1 INTRODUCTION

Esophageal cancer is an aggressive disease with a high degree of both distant and regional metastasis at comparatively early stages.^1^ Neoadjuvant therapy, or adjuvant therapy followed by surgery, is widely used to improve the prognosis of patients with resectable esophageal squamous cell carcinoma (ESCC).^2^, ^3^, ^4^ Neoadjuvant chemoradiotherapy is currently regarded as the standard treatment for patients with resectable esophageal cancer in Western countries.^5^, ^6^, ^7^, ^8^ On the other hand, neoadjuvant chemotherapy such as cisplatin and fluorouracil (CF) chemotherapy is also regarded as an alternative treatment in Asia. However, CF regimen has a low response rate and controversial survival benefit.^9^, ^10^, ^11^, ^12^, ^13^, ^14^

Triplet regimens with Adriamycin, epirubicin, or docetaxel in addition to CF were recently reported to be more effective in patients with advanced esophageal cancer rather than doublet regimen.^15^, ^16^, ^17^, ^18^, ^19^ Thus, we planned a clinical trial of triplet neoadjuvant chemotherapy regimens so that we can select the best chemotherapy regimen in order to compare with chemoradiotherapy in future.

The randomized controlled chemotherapy for esophageal cancer followed by surgery trial (OGSG1003) compared two regimens of neoadjuvant chemotherapy (CF plus Adriamycin (ACF) vs CF plus docetaxel (DCF) plus surgery.^20^ A total of 162 patients at 10 institutions were enrolled between November 2010 and October 2012. The initial results of this trial showed that DCF chemotherapy is associated with prolonged recurrence‐free survival (RFS) compared to ACF.^21^ Here, we report long‐term follow‐up results with analysis of the primary endpoint, RFS, as well as secondary endpoints such as overall survival (OS) and recurrence patterns.

## 2 METHODS

### 2.1 Patients

Full details of the eligibility criteria and pre‐treatment evaluation were reported previously.^14^ Briefly, eligible patients were aged 20 years or older with performance status ≥1, histologically confirmed squamous cell carcinoma of the thoracic esophagus, and adequate bone marrow, renal, hepatic, and pulmonary function. Patients with clinical stage Ib to IIIB, stage IIIC without T4b, or stage IV disease based on only supraclavicular lymph node metastasis were eligible. All patients provided written informed consent before enrolment. The institutional review board at each participating institution approved the study protocol. This study was registered with the University Hospital Medical Information Network Clinical Trials Registry (UMIN‐CTR) of Japan (identification number UMIN000004555/000004616).

### 2.2 Treatment

The randomization scheme was described previously.^14^ Patients were stratified by institution and clinical N stage. They were then randomly assigned to either ACF chemotherapy followed by surgery (ACF group) or DCF chemotherapy followed by surgery (DCF group). ACF chemotherapy consisted of two cycles of Adriamycin (35 mg/m^2^) and cisplatin (70 mg/m^2^) as a 1‐hour intravenous infusion and fluorouracil (5‐FU, 700 mg/m^2^/d) as a continuous intravenous infusion for 7 days (days 1‐7) every 4 weeks. DCF chemotherapy consisted of two cycles of docetaxel (70 mg/m^2^) and cisplatin (70 mg/m^2^) as a 1‐hour intravenous infusion and 5‐FU (700 mg/m^2^/d) as a continuous intravenous infusion for 5 days (days 1‐5) every 3 weeks.

Surgery was scheduled within 6 weeks after completing the last cycle of chemotherapy in both groups. Surgery consisted of subtotal esophagectomy through a right thoracotomy or video‐assisted thoracoscopic surgery. Transhiatal esophagectomy was not performed in this trial. Each surgeon decided on the surgical procedure, including whether cervical lymphadenectomy was added, reconstruction route, and whether organ reconstruction was performed, in accordance with the location of the tumor and institutional practice.

### 2.3 Follow‐up

Patients were seen every 3 months during the first 2 years after the date of random assignment, every 6 months for the next 3 years, and annually thereafter. Disease recurrence was defined as locoregional (at the primary site including the anastomosis, regional lymph nodes, and supraclavicular lymph nodes) or distant (including non‐regional lymph nodes except for the supraclavicular lymph nodes or distant organs). The study protocol stipulated that postoperative adjuvant chemotherapy or chemoradiotherapy was not performed.

### 2.4 Statistical analysis

Data were analyzed according to the intention‐to‐treat principle. OS was measured from the date of randomization to the date of death due to any cause or last follow‐up. RFS was measured from the date of randomization to the date of first evidence of recurrence, death due to any cause, or last follow‐up in patients without recurrence. We used the Kaplan–Meier method to estimate OS and RFS and the log‐rank test to compare differences across groups. Univariate Cox regression modeling was used to analyze differences in recurrence‐free interval between treatment groups. Deaths from non‐disease‐related causes were censored. Recurrence was defined as recurrence in patients with R0 resection and either locoregional residual disease after protocol treatment or distant residual disease during or after protocol treatment in patients with R0 resection or patients who did not undergo resection.

Hazard ratios (HRs) for DCF and ACF and 95% confidence intervals (CIs) were estimated using Cox proportional hazards regression for each subgroup. Differences in OS between the two treatment groups were tested. Subgroups were predefined according to baseline patient characteristics such as age, sex, performance status, location of the primary tumor, clinical T stage, and clinical N stage. Statistical analyses were performed with JMP 13.0.1 (SAS).

## 3 RESULTS

### 3.1 Patient characteristics

**FIGURE 1:**
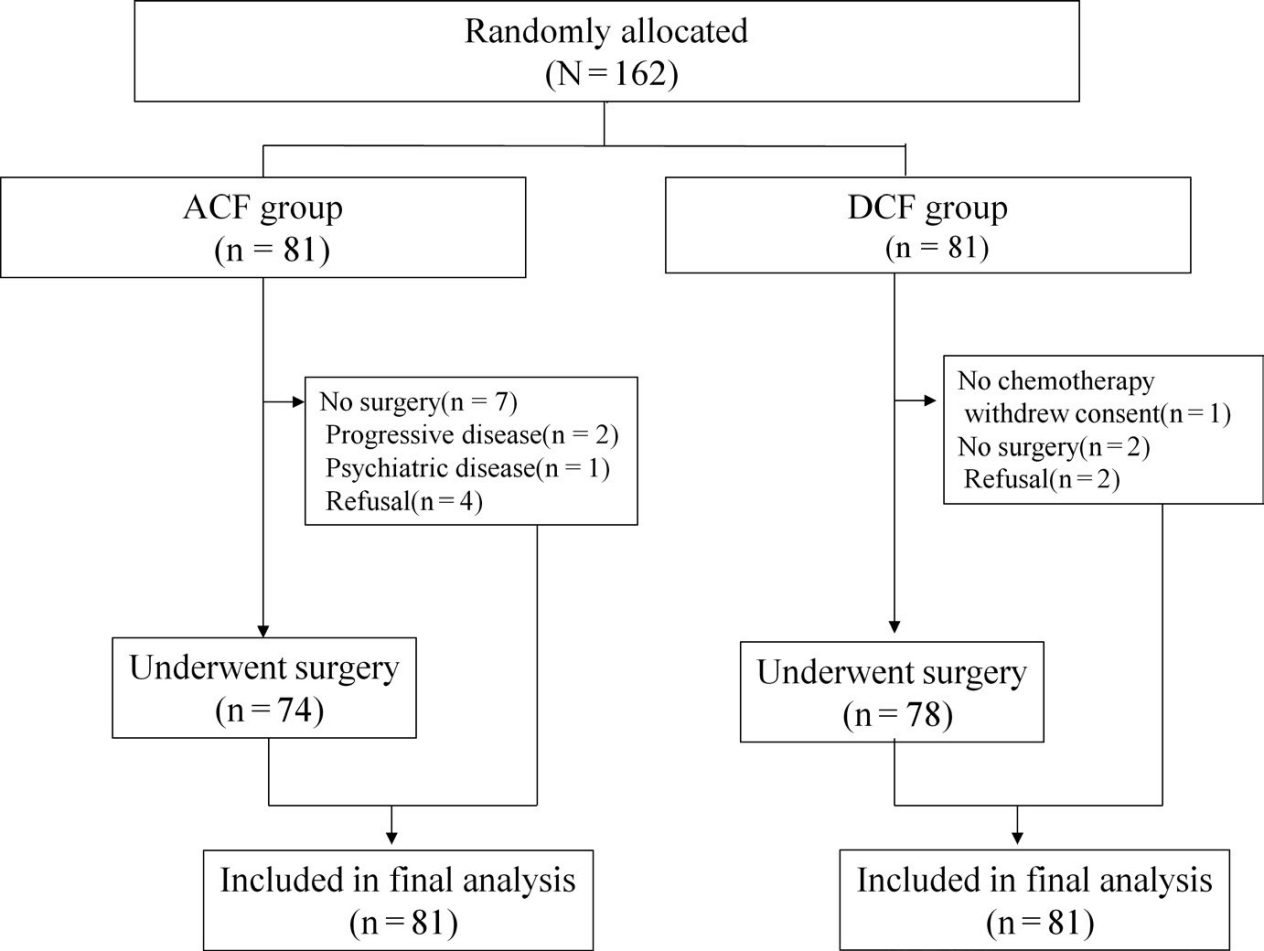
CONSORT diagram

From November 2010 to October 2012, a total of 162 patients with resectable esophageal squamous cell carcinoma were randomly allocated to the two treatment groups in a 1:1 ratio (Figure 1) and included in the intention‐to‐treat analysis. Since the initial reporting of this study, no additional information became available regarding baseline characteristics, which were well‐balanced between the two treatment groups (Table 1). Histological tumor stage and response evaluation are summarized in Table 2.

**Table 1:** Patient characteristics

Characteristic	ACF group (n = 81)	DCF group (n = 81)	*P* value
Age (y)			
Median (range)	68 (46‐79)	65 (44‐78)	.271
Gender			
Male	71	66	.282
Female	10	15	
ECOG performance status			
0	61	63	
1	20	18	
Location			
Upper	7	12	.451
Middle	42	41	
Lower	32	28	
Clinical T stage			
1	1	2	.432
2	17	14	
3	61	65	
4a	2	0	
Clinical N stage			
0	14	17	.942
1	40	39	
2	24	22	
3	3	3	
Clinical M stage			
0	73	73	1.000
1	8	8	
Clinical stage			
IB	4	5	.453
IIA	10	11	
IIB	14	7	
IIIA	22	31	
IIIB	18	16	
IIIC	5	3	
IV	8	8	
Histopathological grade			
G1	11	8	.821
G2	32	31	
G3	7	6	
Gx	31	36	

Abbreviations: ACF, adriamycin + cisplatin and fluorouracil; DCF, docetaxel + cisplatin and fluorouracil; ECOG, Eastern Cooperative Oncology Group.

**Table 2:** Histopathological tumor response and stage

	ACF group (n = 73)	DCF group (n = 78)	*P* value
pT stage			
0	0 (0%)	11 (14%)	.008
1	16 (22%)	17 (22%)	
2	18 (25%)	13 (17%)	
3	34 (47%)	33 (42%)	
4	5 (7%)	3 (5%)	
pN stage			
0	27 (37%)	33 (42%)	.367
1	25 (34%)	20 (26%)	
2	14 (19%)	21 (27%)	
3	7 (10%)	4 (5%)	
pM stage			
0	61 (84%)	72 (92%)	.092
1	12 (16%)	6 (8%)	
pStage			
0	0 (0%)	7 (9%)	.015
I	16 (22%)	14 (18%)	
II	16 (22%)	21 (27%)	
III	29 (40%)	30 (39%)	
IV	12 (16%)	6 (8%)	
Residual tumor			
R0	70 (96%)	75 (96%)	.932
R1/2	3 (4%)	3 (4%)	
Histopathological tumor response			
0	11 (15%)	2 (3%)	<.001
1a	35 (48%)	22 (28%)	
1b	14 (19%)	21 (27%)	
2	13 (18%)	22 (28%)	
3	0 (0%)	11 (14%)	

Abbreviations: ACF, adriamycin + cisplatin and fluorouracil; DCF, docetaxel + cisplatin and fluorouracil.

The final day of follow‐up was January 2018, guaranteeing a minimum follow‐up of 5 years for all patients included in the analysis except for one patient in the DCF group who was lost to follow‐up after moving at 26 months after randomization. The median follow‐up for surviving patients was 69.8 months.

### 3.2 Recurrence pattern

Of 81 eligible patients randomly assigned to the DCF group, 78 underwent surgery: 75 (93%) patients underwent R0 resection and three (3.7%) underwent R1/2 resection. Among the 81 patients randomly assigned to the ACF group, 74 underwent surgery: 70 (86%) patients underwent R0 resection, three (3.7%) patients underwent R1/2 resection, and one (1.2%) patient underwent exploratory thoracotomy.

Table 3 presents the pattern of first recurrence in patients who underwent R0 resection or pattern of residual disease in patients who underwent R1/2 resection or those who did not undergo surgery. Recurrence occurred in 76 patients (47%). Four patients experienced recurrence since the previous report. In the DCF group, 29 patients had recurrence or residual disease: 18 had locoregional disease, 17 had distant disease, and six had both locoregional and distant disease. In the ACF group, 47 patients had recurrence or residual disease: 29 had locoregional disease, 26 had distant disease, and eight had both locoregional and distant disease. Compared to the ACF group, the DCF group had significantly higher control rates for locoregional and distant disease.

**Table 3:** Patterns of recurrence or residual disease by treatment group

	All	ACF group (n = 81)	DCF group (n = 81)	*P* value
Overall	76	47 (58%)	29 (36%)	.004
Locoregional	47	29 (36%)	18 (22%)	.030
Distant	43	26 (32%)	17 (21%)	.041
Lymph node	8	6 (7%)	2 (2%)	.277
Liver	11	8 (10%)	3 (4%)	.210
Lung	10	3 (4%)	7 (9%)	.328
Bone	6	4 (5%)	2 (2%)	.681
Brain	1	1 (1%)	0 (0%)	1.000
Pleura	11	7 (9%)	4 (5%)	.534
Other	5	2 (3%)	3 (4%)	1.000

Abbreviations: ACF, Adriamycin + cisplatin and fluorouracil; DCF, docetaxel + cisplatin and fluorouracil.

After recurrence, 37 patients (79%) had treatment in the ACF group while 25 patients (86%) had treatment in the DCF group (*P* = .547). As first treatment after recurrence, 16 patients (34%) had chemotherapy, 14 (30%) had chemoradiotherapy, five (11%) had radiotherapy, and two (4%) had surgery in the ACF group. On the other hand, 10 patients (34%) had chemoradiotherapy, nine (31%) had chemotherapy, four (14%) had radiotherapy, and two (7%) had surgery in the DCF group. The difference in the first treatment after recurrence difference was not seen between two groups (*P* = .934).

### 3.3 Survival

**FIGURE 2:**
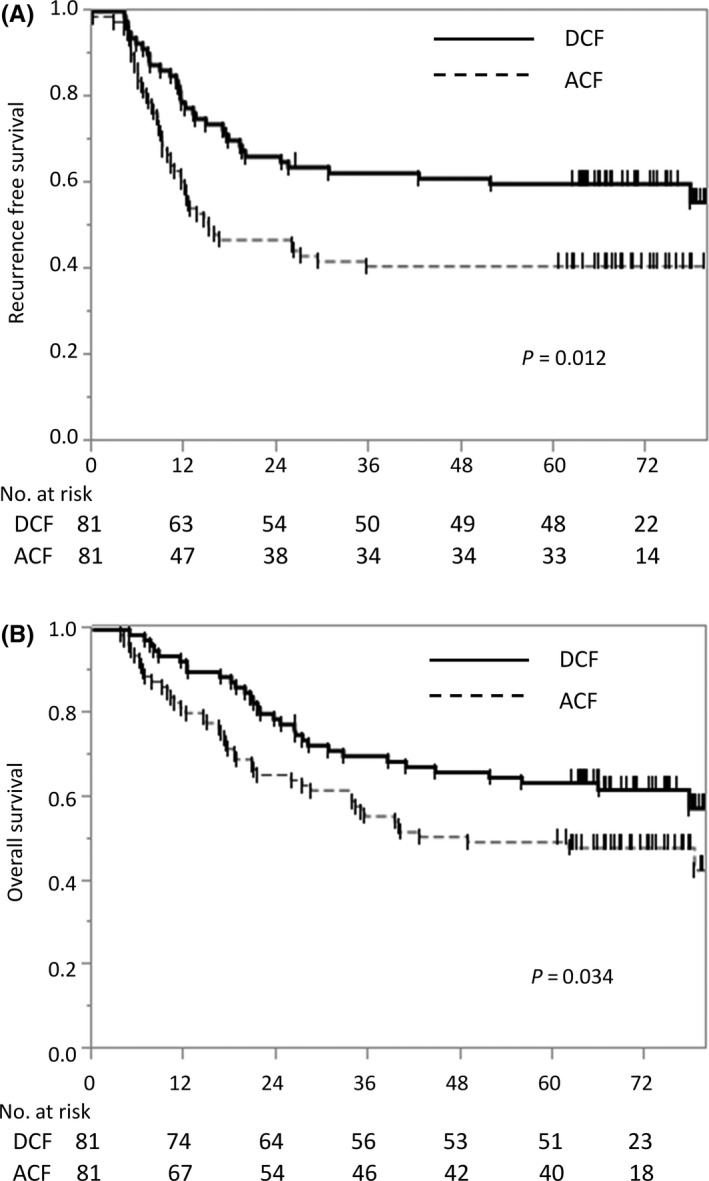
Kaplan‐Meier estimates of (A) recurrence‐free and (B) overall survival. A, The 5‐year recurrence‐free survival (RFS) was 40.7% in the Adriamycin + cisplatin and fluorouracil (ACF) group and 59.9% in the cisplatin and fluorouracil plus docetaxel (DCF) group (HR, 0.55; 95% CI, 0.35‐0.86; *P* = .012). B, The 5‐year OS was 49.4% in the ACF group and 63.5% in the DCF group (HR, 0.61; 95% CI, 0.38‐0.96; *P* = .034)

At the time of this analysis, the median follow‐up was 61 months in the ACF group and 65 months in the DCF group. The 5‐year RFS was 40.7% in the ACF group and 59.9% in the DCF group (Figure 2A). Compared with the ACF group, the DCF group had a significantly higher likelihood of RFS (HR, 0.55; 95% CI, 0.35‐0.86; *P* = .012). The 5‐year OS was 49.4% in the ACF group and 63.5% in the DCF group (Figure 2B). Compared with the ACF group, the DCF group also had a significantly higher likelihood of OS (HR, 0.61; 95% CI, 0.38‐0.96; *P* = .034).

**FIGURE 3:**
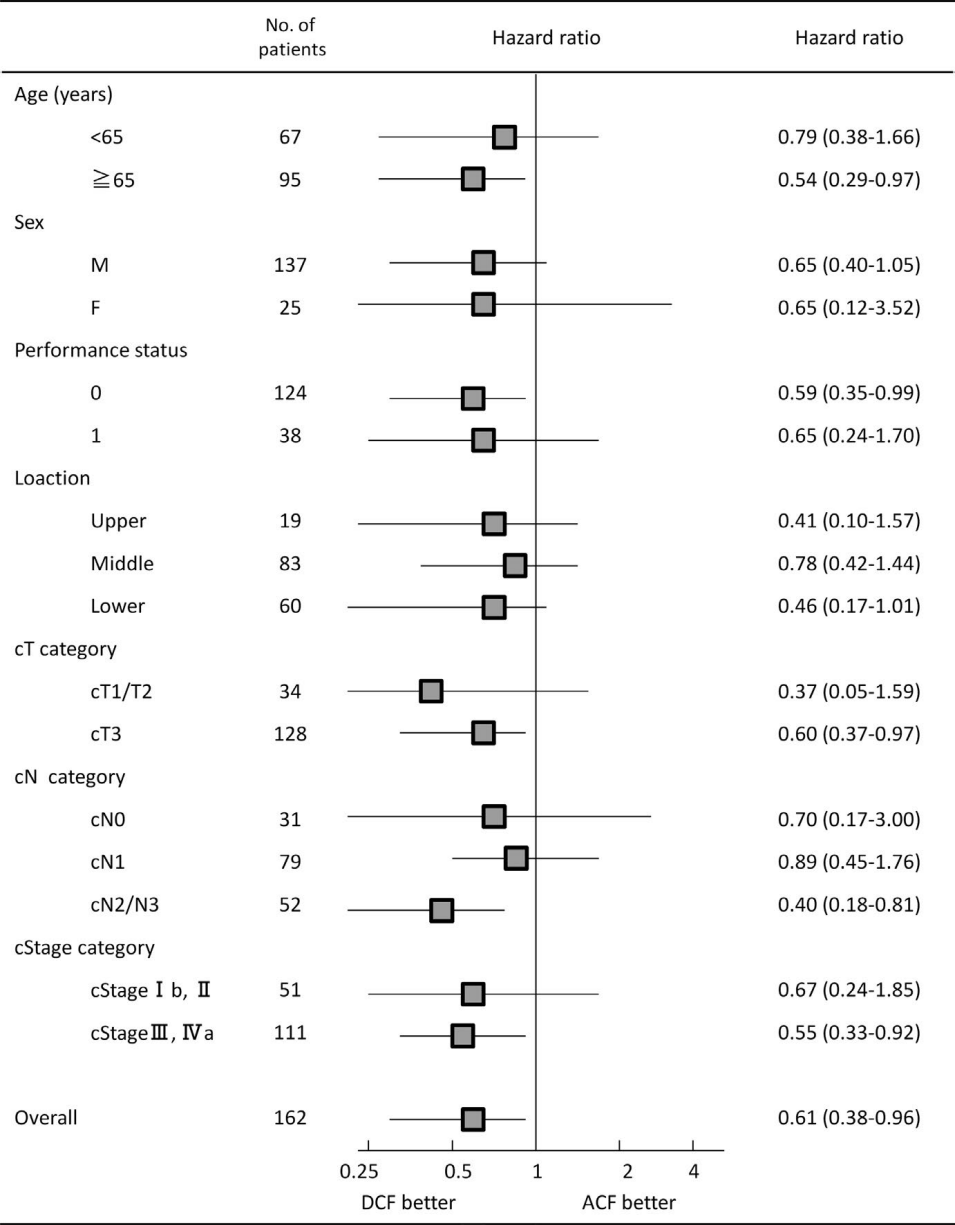
Forest plot of hazard ratios (HRs) and 95% confidence intervals (CIs) for survival in 162 patients with esophageal cancer stratified by baseline characteristics

We found no differences in OS between patients receiving ACF vs DCF by age, sex, performance status, and tumor location (Figure 3). On the other hand, the OS of patients with advanced clinical T (cT3 or cT4) stage and N (cN2 or cN3) stage who underwent DCF was significantly better than those who underwent ACF. However, we found no differences in OS between the two groups among those with early clinical T (cT1 or cT2) stage and N (cN0 or cN1) stage.

**FIGURE 4:**
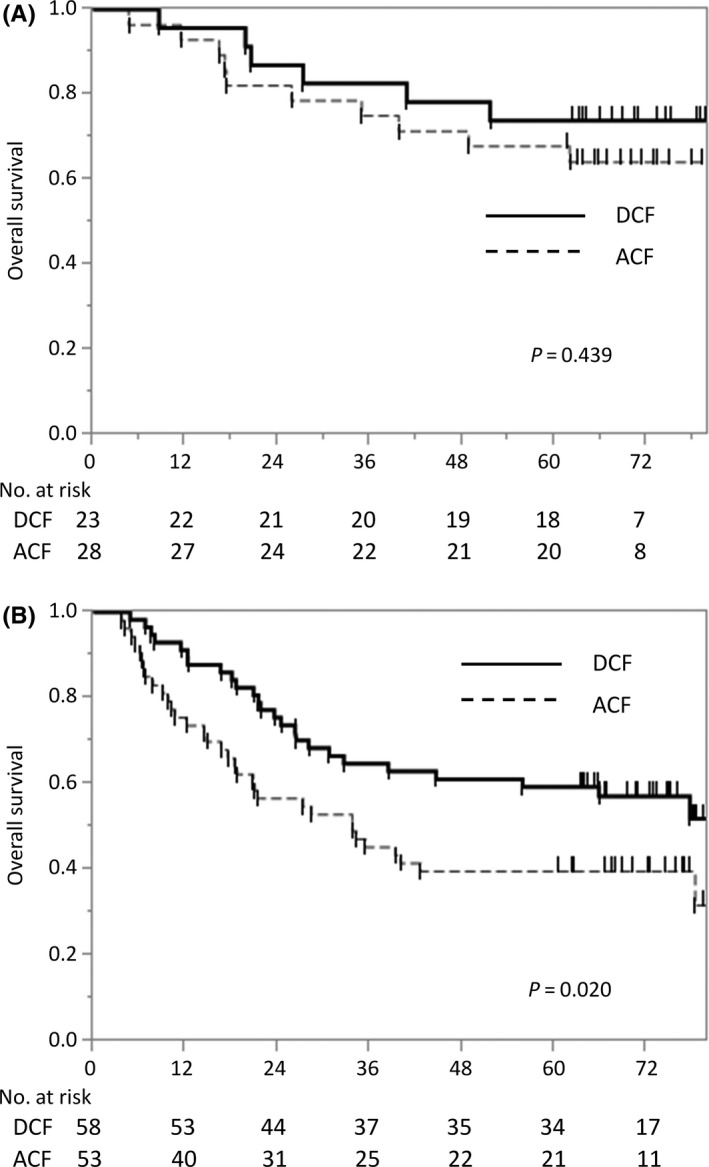
Kaplan‐Meier estimates of overall survival in patients with (A) cStage I or II disease and (B) cStage III or IV disease. A, In patients with cStage I or II disease, the 5‐year OS was 67.9% in the Adriamycin + cisplatin and fluorouracil (ACF) group and 73.9% in the cisplatin and fluorouracil plus docetaxel (DCF) group (*P* = .439). B, In patients with cStage III or IV disease, the 5‐year OS was 39.6% in the ACF group and 59.3% in the DCF group (HR, 0.55; 95% CI, 0.33‐0.92;*P* = .020)

Figure 4 presents the OS curves for patients with cStage I or II (Figure 4A) and cStage III or IV (Figure 4B) cancer on the basis of preoperative chemotherapy regimen (ACF vs DCF). In patients with cStage I or II disease, the 5‐year OS was 67.9% in the ACF group and 73.9% in the DCF group (*P* = .439). In patients with cStage III or IV disease, the 5‐year OS was 39.6% in the ACF group and 59.3% in the DCF group. Patients who underwent preoperative DCF followed by surgery had significantly better survival than those who underwent preoperative ACF (HR, 0.55; 95% CI, 0.33‐0.92; *P* = .020).

## 4 DISCUSSION

These long‐term results confirm the initial report that preoperative DCF chemotherapy followed by surgery is associated with prolonged RFS when compared to preoperative ACF chemotherapy followed by surgery. The present results also show that compared with preoperative ACF chemotherapy, DCF chemotherapy improves OS when followed by surgery. Furthermore, there was significantly less locoregional and distant recurrence in the DCF group than in the ACF group. The DCF regimen may be a candidate neoadjuvant therapy for resectable ESCC.

The present study showed that neoadjuvant DCF is superior to neoadjuvant ACF in terms of RFS and OS. To the best of our knowledge, this is the first prospective comparison of superiority for preoperative chemotherapy regimens for esophageal cancer in terms of long‐term prognosis. To date, one report has compared prospective preoperative chemotherapy regimens for esophageal cancer. Alderson et al^22^ compared preoperative cisplatin plus 5‐FU with epirubicin, cisplatin, and capecitabine (ECX) followed by surgery in patients with esophageal adenocarcinoma in the phase 3 UK MRC OE05 trial. They showed that the ECX group had a higher pathological treatment effect for primary esophageal lesions and a higher pN0 rate. However, they also showed that the median survival was 23.4 months in the CF group and 26.1 months in the ECX group, which was similar. Therefore, they concluded that preoperative ECX does not prolong survival compared with preoperative CF. The differences between their results and our results might be due to differences in the rate of curative resection as well as histological type. Indeed, in UK MRC OE05, the ECX group had a more pathological effect rather than CF group, but the proportion of patients with Mandard 1‐2 disease was as low as 17%. Among the 446 patients assigned to the ECX group, R0 resection was possible in 223 patients, only 50% of the total. On the other hand, the R0 resection rate in the DCF group in this study was 93%, which may indicate that chemotherapy was more likely to lead to a difference in resectability.

The subgroup analysis of this study showed that DCF is superior to ACF in terms OS in patients with clinical Stage 3 or 4 disease, but DCF and ACF had similar effects in patients with clinical stage 1 or 2 disease. This might be partly because there was a difference in clinical response between patients with clinical stage 1 or 2 vs 3 or 4 disease. Indeed, in patients with stage 1 or 2 disease, the response rates for ECF and DCF were equivalent at 71% and 74%, respectively, whereas in patients with stage 3 or 4 disease, the response rates for ECF and DCF were 45% and 68%, respectively. Based on these points mentioned above, DCF is a good candidate for preoperative chemotherapy for stage 3 and 4 disease, but a slightly less toxic regimen may be considered for stage 1 and 2 disease from the viewpoint of toxicity.

The present study revealed that the DCF group had less postoperative local recurrence than the ACF group. This can be explained by the fact that DCF has a stronger effect on primary tumors than ACF. In fact, the pathological treatment effect was 17.8% for ACF vs 42.3% for DCF in patients with grade ≥2 disease. Pathological T stage was also significantly lower in the DCF group than in the ACF group. Our results were consistent with previous results. Oppedijk et al^8^ analyzed the pattern of recurrence after preoperative chemoradiation followed by surgery in the CROSS trial. Their results suggested that local relapse was less likely to occur when the response to preoperative chemoradiotherapy is more effective.

This study also indicated that preoperative DCF is significantly more effective than ACF in preventing distant metastatic recurrence. Although individual recurrence sites were similar, recurrences in distant lymph nodes and liver and metastases in the pleura tended to be smaller in the DCF group. This result indicates that preoperative chemotherapy regimens with higher clinical response and pathological effect are more beneficial in suppressing recurrence of distant metastasis.

In the present study, recurrence occurred in 76 patients. Fifty‐two (82%) of the recurrent patients had treatment. First treatment after recurrence between two groups was similar. Additionally, after recurrence, the rates of use of regimens containing docetaxel were similar in both groups (38% vs 31%). Thus, in this study, contents of preoperative treatment did not affect choice of treatment after recurrence.

This study has several limitations. This was a phase 2 trial with only a small number of patients. Thus, the results cannot be used to determine standards for preoperative treatment. The NExT trial (JCOG1109), a randomized controlled trial of preoperative chemoradiotherapy and chemotherapy, is currently ongoing in Japan. The results will provide evidence of the most appropriate preoperative treatment for ESCC.^23^

In conclusion, this study showed that, compared to ACF chemotherapy, DCF chemotherapy is associated with prolonged RFS and OS for patients with resectable ESCC. Thus, DCF chemotherapy may be a candidate neoadjuvant therapy for resectable ESCC.

## DISCLOSURE

Funding: The authors report no funding sources for any products mentioned or concepts discussed in this article.

Conflict of Interest: The authors declare no conflicts of interest.

Author Contribution: Conception and design: T Yasuda, M Yano, Y Doki. Development of the methodology: K sugimura, M Yamasaki, M Hirao, K Fijitani, Y Kimura, H Miyata, M Motoori, A Takeno, O Shiraishi, T Makino, T Kii, K Tanaka, T Satoh, M Mori. Acquisition of data: K sugimura, M Yamasaki, M Hirao, K Fijitani, Y Kimura, H Miyata, M Motoori, A Takeno, O Shiraishi, T Makino, T Kii, K Tanaka, T Satoh, M Mori. Analysis and interpretation of data: K sugimura, M Yamasaki, T Yasuda, M Yano, M Hirao, K Fijitani, Y Kimura, H Miyata, M Motoori, A Takeno, O Shiraishi, T Makino, T Kii, K Tanaka, T Satoh, M Mori, Y Doki. Writing, review, and revision of the manuscript: K sugimura, M Yamasaki.

## ETHICS APPROVAL AND CONCENT TO PARTICIPATE

The Human Ethics Review Committee of each institute approved the study protocol. Subjects have provided written informed consent. This study was performed in accordance with the Declarations of Helsinki.

## CONSENT FOR PUBLICATION

All the authors declare consent for publication.

## Data Availability

The authors declare that the data supporting the findings of this study are available within the article.
